# Impact of signal-averaged electrocardiography findings on appropriate shocks in prophylactic implantable cardioverter defibrillator patients with nonischemic systolic heart failure

**DOI:** 10.1186/s12872-022-02811-6

**Published:** 2022-08-16

**Authors:** Michiru Nomoto, Atsushi Suzuki, Tsuyoshi Shiga, Morio Shoda, Nobuhisa Hagiwara

**Affiliations:** 1 Department of Cardiology, Tokyo Women’s Medical University, Tokyo, Japan; 2 Department of Clinical Pharmacology and Therapeutics, The Jikei University School of Medicine, 3-25-8 Nishi-shinbashi, Minato-ku, Tokyo, 105-8461 Japan; 3 Clinical Research Division for Heart Rhythm Management, Tokyo Women’s Medical University, Tokyo, Japan

**Keywords:** Heart failure, Implantable cardioverter defibrillator, Nonischemic, Shocks, Signal-averaged electrocardiogram

## Abstract

**Background:**

Appropriate shock therapy is associated with subsequent all-cause death in heart failure (HF) patients who receive an implantable cardioverter defibrillator (ICD) for the primary prevention of sudden cardiac death. To evaluate the impact of signal-averaged electrocardiography (SAECG) findings on appropriate shocks in prophylactic ICD patients with nonischemic systolic HF.

**Methods:**

We studied 86 patients with nonischemic HF and a left ventricular ejection fraction ≤ 35% who underwent new ICD implantation for the primary prevention of sudden cardiac death. We excluded patients who had a previously implanted permanent pacemaker and patients who received cardiac resynchronization therapy with an ICD. SAECG was performed before implantation. Abnormal SAECG findings were defined if 2 of the following 3 conditions were identified: filtered QRS duration (fQRS) ≥ 114 ms, root-mean-square voltage during the last 40 ms of the fQRS (RMS 40) < 20 μV, and duration of the low-amplitude potentials < 40 μV (LAS 40) > 38 ms; additionally, patients with a QRS complex ≥ 120 ms who met both the RMS 40 and LAS 40 criteria were also considered to have abnormal SAECG findings. The primary outcome was the first occurrence of appropriate shock after implantation of the ICD. The secondary outcomes were the first occurrence of inappropriate shock and all-cause mortality.

**Results:**

Forty-two patients met the criteria for abnormal SAECG findings (49%). During a median follow-up period of 61 months, 17 patients (20%) died, 24 (28%) received appropriate shock therapy, and 19 (22%) received inappropriate shock therapy. There was a significantly higher incidence of appropriate shocks in patients with abnormal SAECG findings than in those with normal SAECG findings (log-rank test, *p* = 0.025). Multivariate analysis revealed that abnormal SAECG findings were independently associated with the occurrence of appropriate shock (hazard ratio 2.67, 95% confidential interval 1.14–6.26). However, abnormal SAECG findings were not related to inappropriate shock. There was no difference in the incidence of all-cause death between patients with abnormal and normal SAECG findings.

**Conclusions:**

Our results suggest that abnormal SAECG findings are associated with a high probability of appropriate shocks in prophylactic ICD patients with nonischemic systolic HF.

**Supplementary Information:**

The online version contains supplementary material available at 10.1186/s12872-022-02811-6.

## Background

Implantable cardioverter defibrillators (ICDs) are a potential treatment for preventing sudden cardiac death (SCD) in patients with low left ventricular ejection fraction (LVEF) and myocardial infarction/heart failure (HF), and ICDs have been shown to reduce the occurrence of SCD and improve prognosis [1–3]. However, appropriate shock therapy is also reported to be associated with subsequent all-cause death in patients who receive an ICD for the primary prevention of SCD [4–7]. Moreover, ICD shocks impair patients’ quality of life and psychological status [8]. Recently, the EUropean Comparative Effectiveness Research to Assess the Use of Primary ProphylacTic Implantable Cardioverter-Defibrillators (EU-CERT-ICD) showed in a large cohort that digitalis use, male sex, chronic obstructive pulmonary disease and increased QT_C_ (per 40 ms) were risk factors for appropriate shock in primary prevention ICD patients [9]. Nevertheless, potent predictors of appropriate shock therapy for patients with HF and reduced LVEF with primary prophylactic ICDs have yet to be determined.

Signal-averaged electrocardiography (SAECG) is a high-resolution surface electrocardiography technique that detects low-amplitude waveforms and late potentials within the terminal portion of QRS waves. Abnormal SAECG findings can predict arrhythmic substrates that lead to the development of ventricular tachyarrhythmias and is related to outcomes in cases of infarct [10]. In general, cases of ischemic etiology might be driven, at least in part, by the induction of ischemic events. In patients with nonischemic dilated cardiomyopathy, abnormal SAECG findings predict ventricular tachyarrhythmias, SCD or all-cause mortality, although controversy exists [10–13]. Abnormal SAECG findings alone have a low positive predictive value for sustained ventricular tachycardia (VT)/fibrillation (VF) and SCD among patients with a wide range of cardiovascular diseases [10]. However, SAECG may be useful for the risk stratification of appropriate shocks among selected patients with nonischemic HF at high risk for SCD. Therefore, this study aimed to evaluate the impact of abnormal SAECG findings on appropriate shocks in nonischemic systolic HF patients who received an ICD for the primary prevention of SCD.

## Methods

### Patients

All patients with nonischemic HF and an LVEF ≤ 35% who prophylactically underwent new implantation of an ICD device from January 2000 to May 2018 at Tokyo Women’s Medical University Hospital were retrospectively included in this study. We excluded patients who received ICD devices for secondary prevention of SCD due to a history of sustained VT/VF and survivors of SCD. We also excluded patients who had a previously implanted pacemaker and patients with cardiac resynchronizing therapy (biventricular ICDs) from our analysis. A nonischemic etiology was defined as the absence of coronary artery disease, as confirmed by coronary angiography. LVEF was measured using the modified Simpson’s method by echocardiography. Indications for the implantation of an ICD device were chronic HF with a New York Association (NYHA) functional class II/III and LVEF ≤ 35% despite appropriate medical treatment according to the guidelines of the American College of Cardiology Foundation and American Heart Association or Japanese Circulation Society [14, 15].

This study categorized the native QRS complex as follows. A narrow QRS was defined as ≤ 100 ms. Left bundle branch block (LBBB) and right bundle branch block (RBBB) were defined according to present definitions with a QRS duration > 120 ms. Intraventricular conduction delay (IVCD) was defined as a QRS duration 101–120 ms regardless of morphology or a QRS duration > 120 ms that did not meet the classical bundle branch block definitions and was further divided into IVCD with LBBB-predominant (L-IVCD) and non-LBBB-predominant (O-IVCD) features. L-IVCD required the following 12-lead electrocardiographic findings: net negative in lead V1, no terminal positivity in lead V1, and net positive in lead I [16].

SAECG was performed within 2 weeks before ICD implantation in all patients. SAECG was performed when the patients were in stable condition without the use of intravenous diuretics, inotropes and/or vasodilators and without mechanical circulatory support. This study was approved by the institutional review board of Tokyo Women’s Medical University.

### Outcomes

The primary outcome was the first occurrence of an appropriate shock after ICD implantation. The secondary outcomes were the first occurrence of an inappropriate shock and all-cause mortality. Data for VT/VF occurrence requiring ICD shock were obtained by reviewing the event details and electrograms stored on ICD disks.

The VF detection zone was programmed for all patients. The VT zone was programmed for 72 patients; 2 VT zones were programmed for 6 of these patients. ICD shock (defibrillation) occurred when ventricular arrhythmias were detected in the VF zone or the fast VT zone (in some cases). Antitachycardia pacing (ATP), including burst and/or autodecremental ramp pacing, was delivered when triggered by VT. A shock was delivered if the pacing failed to terminate the VT. Delayed high-rate programming has been used to avoid unnecessary shocks according to the guidelines [17] and its use significantly increased over the years of the study period (Additional file 1: Table S1).

### Follow-up

**Fig. 1:**
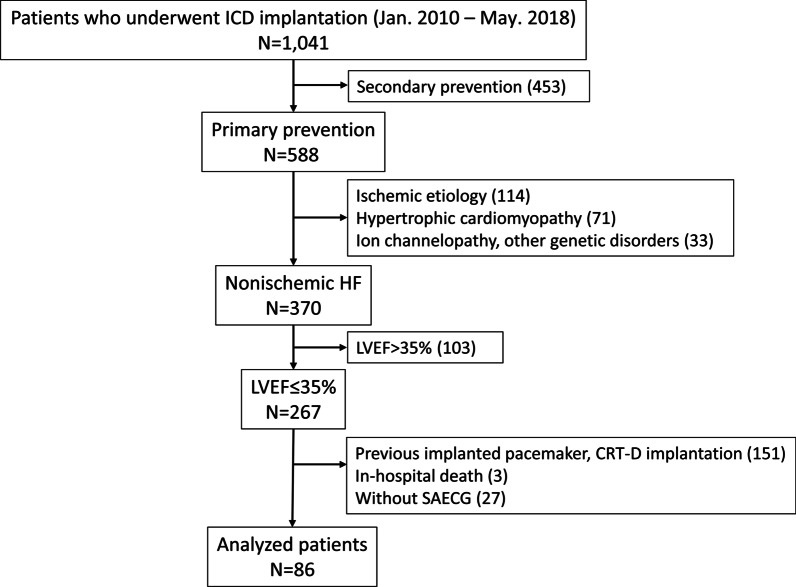
Patient flow chart for this study. CRT-D, cardiac resynchronization therapy with a defibrillator; HF, heart failure; ICD, implantable cardioverter defibrillator; LVEF, left ventricular ejection fraction; SAECG, signal-averaged electrocardiography

Follow-up was performed every 3–6 months up to December 2021 at our ICD clinic. The patients were followed until death from any cause, a change in ICD clinic, loss to follow-up, or December 2021. Information regarding the deceased patients was obtained from medical records, family members, the patients’ general practitioners, and the hospitals to which the patients had been admitted. Eleven patients (13%) were followed for over 5 months after ICD implantation at our hospital but were subsequently referred to the ICD clinics of other hospitals. One patient was lost to follow-up (Fig. 1).

#### SAECG

SAECG (Predictor BSM-32, Arrhythmia Research Technology, Fitchburg, Massachusetts, USA) findings were recorded with standard X, Y, and Z orthogonal leads using high-pass filtering. The signals from the leads were combined into a vector magnitude (the root sum square of the signals of each lead). Approximately 200 beats were averaged to obtain a noise level of < 0.5 μV. The following parameters were calculated: the filtered QRS duration (fQRS), root-mean-square voltage during the last 40 ms of the fQRS (RMS 40), and duration of low-amplitude potentials < 40 μV (LAS 40). Abnormal SAECG findings were defined if 2 of the following 3 parameter conditions were observed: fQRS ≥ 114 ms, RMS 40 < 20 μV, and LAS 40 > 38 ms [18]; additionally, the combination of RMS 40 < 20 μV and LAS 40 > 38 ms indicated abnormal findings for patients with a QRS complex ≥ 120 ms.

### Statistical analysis

Data are presented as medians with interquartile ranges or number of patients (percentage). Baseline clinical and electrocardiographic data were compared between groups with abnormal and normal SAECG findings using the Mann–Whitney U test. Categorical variables were subjected to chi-squared analysis. Cumulative proportions of the event-free rate were calculated using the Kaplan–Meier method, and differences in event-free rates were compared using the log-rank test. Univariate Cox regression analysis was applied to estimate the relationship between SAECG findings and appropriate or inappropriate shocks. Multivariate analyses using the Cox proportional hazards model were performed to assess predictors of appropriate shocks from the following variables and abnormal SAECG findings: age, male sex, LVEF ≤ 30%, NYHA functional class II, estimated glomerular filtration rate (eGFR) according to the Modification of Diet in Renal Disease formula < 60 ml/min/1.73 m^2^, atrial fibrillation, beta-blocker use, digoxin use, and amiodarone use. The forward stepwise method was used for the multivariate analyses with a p value threshold set at 0.05. Data analyses were performed with IBM SPSS statistical software (version 22.0, IBM corporation, Armonk, NY, USA).

## Results

The baseline clinical characteristics of the patients are summarized in Table 1. Forty-two patients met the criteria for abnormal SAECG findings (49%). The QRS duration was longer in patients with abnormal SAECG findings than in those with normal SAECG findings, and a QRS duration ≥ 120 ms was more frequent in patients with abnormal SAECG findings than in those with normal SAECG findings. There were no differences in the types of QRS complexes between patients with and without abnormal SAECG findings. Patients with abnormal SAECG findings were likely to use digoxin and amiodarone.

**Table 1:** Patient characteristics

Variables	Normal SAECG	Abnormal SAECG	*P*-value
	(n = 44)	(n = 42)	
Age, years	57 [40–67]	55 [43–66]	0.653
Men, n (%)	30 (68)	32 (76)	0.408
Systolic blood pressure, mmHg	106 [98–122]	102 [96–120]	0.838
Diastolic blood pressure, mmHg	60 [56–68]	60 [54–70]	0.865
Body mass index, kg/m^2^	23.4 [19.6–24.9]	21.1 [19.3–25.1]	0.534
LVEF, %	27 [23–32]	26 [19–30]	0.079
Nonsustained VT, n (%)	42 (98)	41 (98)	0.987
Underlying heart disease			0.390
Idiopathic dilated cardiomyopathy	35 (80)	34 (80)	
End-stage hypertrophic cardiomyopathy	1 (2)	0 (0)	
Valvular heart disease	0 (0)	2 (5)	
Cardiac sarcoidosis	1 (2)	2 (5)	
Arrhythmogenic right ventricular cardiomyopathy	0 (0)	2 (5)	
Congenital heart disease	1 (2)	1 (2)	
Others	6 (14)	1 (2)	
NYHA functional class II/III/IV	39/4/1	38/4/0	0.617
Plasma BNP, pg/mL	211 [98–502]	223 [98–492]	0.986
eGFR, mL/min/1.73 m^2^	80 [49–105]	80 [62–96]	0.873
12-lead electrocardiography			
Atrial fibrillation	7 (16)	10 (24)	0.358
Heart Rate, bpm	71 [65–77]	65 [59–79]	0.289
QTc, ms	447 [418–480]	434 [400–461]	0.171
QRS duration, ms	103 [95–110]	111 [98–121]	0.049
QRS ≥ 120 ms	3 (7)	14 (33)	0.004
Types of QRS complexes			0.401
Narrow (≤ 100 ms)	19 (43)	15 (36)	
L-IVCD	17 (39)	13 (31)	
O-IVCD	6 (14)	7 (17)	
LBBB	0	1 (2)	
RBBB	2 (5)	6 (14)	
Medications			
Beta-blockers	39 (89)	37 (88)	0.938
ACE inhibitors/ARBs	41 (93)	39 (93)	0.953
MRAs	30 (68)	27 (64)	0.702
Digoxin	9 (20)	21 (50)	0.004
Amiodarone	17 (39)	25 (60)	0.053

Values are number (%) or median [interquartile range]

*ACE* angiotensin-converting enzyme; *ARB* angiotensin II receptor blocker; *BNP* B-type natriuretic peptide; *eGFR* estimated glomerular filtration rate; *LBBB* left bundle branch block; *L-IVCD* intraventricular conduction delay with left ventricular branch block-predominant feature; *LVEF* left ventricular ejection fraction; *MRA* mineralocorticoid receptor antagonist; *NYHA* New York Heart Association; *O-IVCD* intraventricular conduction delay with non-left bundle branch block-predominant feature; *QTc* corrected QT interval; *RBBB* right bundle branch block; *SAECG* signal-averaged electrocardiography; *VT* ventricular tachycardia

During a median follow-up period of 61 (36–94) months, 17 patients (20%) died (8 patients with normal SAECG findings and 9 patients with abnormal SAECG findings), 24 (28%) received appropriate shock therapy (6 patients with normal SAECG findings and 16 patients with abnormal SAECG findings), and 19 (22%) received inappropriate shock therapy (8 patients with normal SAECG findings and 11 patients with abnormal SAECG findings). Detailed numbers of appropriate ATPs/shocks in patients who received ICD therapies are listed in Additional file 1: Table S2. Appropriate ICD shocks were less frequent than appropriate ATPs for terminating ventricular tachyarrhythmias in both patients with normal and patients with abnormal SAECG findings. The median numbers of appropriate shocks per patient was 4 and 2 in patients with normal and abnormal SAECG findings, respectively, and the median numbers of inappropriate shocks per patient was 1 and 3 in patients with normal and abnormal SAECG findings, respectively (Additional file 1: Table S3).

**Fig. 2:**
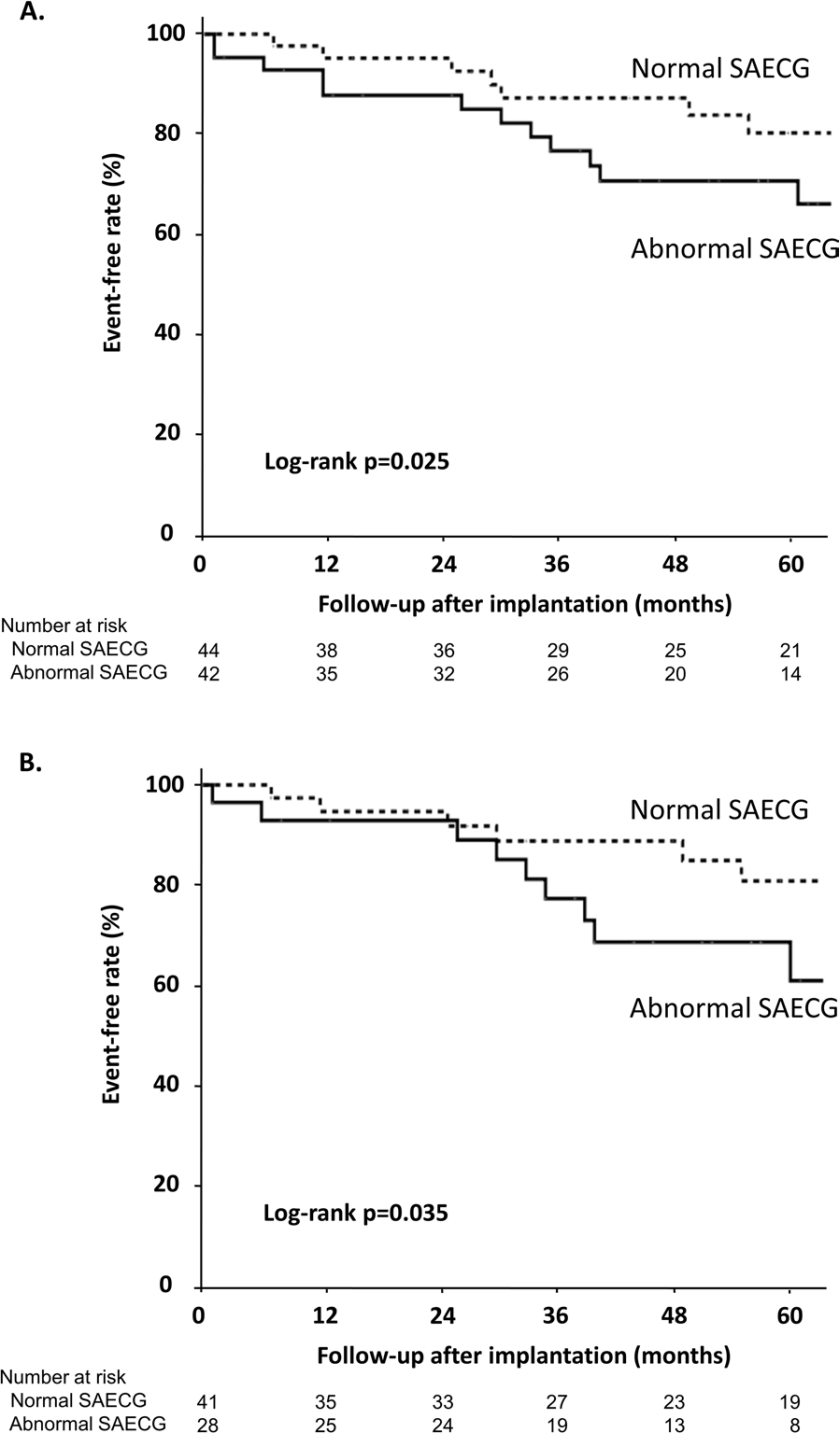
Kaplan–Meier curves for the time to first appropriate ICD shock in nonischemic systolic HF patients who received an ICD with abnormal SAECG findings and normal SAECG findings (**A**) and patients with a QRS complex < 120 ms among the patients in A (**B**)

Kaplan–Meier curves for the time to first appropriate ICD shock are shown in Fig. 2. There was a significantly higher incidence of appropriate shocks in patients with abnormal SAECG findings than in patients with normal SAECG findings, even when limited to patients with a QRS complex < 120 ms. Among 17 patients with a QRS complex ≥ 120 ms, 14 (82%) patients met both the RMS 40 and LAS 40 criteria, and 7 of these 14 patients with abnormal SAECG findings experienced appropriate shocks, while 1 of 3 patients without abnormal SAECG findings experienced appropriate shocks. Univariate analysis for SAECG findings and appropriate shocks showed that abnormal SAECG findings were significantly related to appropriate ICD shocks. For each item of SAECG, fQRS ≥ 114 ms, RMS 40 < 20 μV and LAS > 38 ms were significantly related to appropriate ICD shocks. However, abnormal SAECG findings were not related to inappropriate ICD shocks (Table 2). Multivariate analysis revealed that abnormal SAECG findings and an eGFR < 60 mL/min/1.73 m^2^ were high-risk factors for appropriate shocks (Table 3).

**Table 2:** Abnormal SAECG and ICD shocks

	HR	95% CI	*P*-value
*Appropriate shocks*			
Abnormal SAECG	2.56	1.09–6.01	0.031
fQRS ≥ 114 ms*	4.33	1.24–15.11	0.021
RMS 40 < 20 μV	2.60	1.11–6.11	0.028
LAS 40 > 38 ms	2.39	1.02–5.61	0.044
*Inappropriate shocks*			
Abnormal SAECG	1.52	0.61–3.77	0.372
fQRS ≥ 114 ms*	0.51	0.17–1.53	0.233
RMS 40 < 20 μV	1.21	0.49–2.99	0.674
LAS 40 > 38 ms	1.45	0.58–3.62	0.422

*CI* confidence interval; *fQRS* filtered QRS duration; *HR* hazard ratio; *LAS* 40 duration of low-amplitude potentials < 40 μV; *RMS* 40 root-mean-square voltage in the last 40 ms of the fQRS; *SAECG* signal-averaged electrocardiogram

*Patients with QRS complex < 120 ms

**Table 3:** Univariate and multivariate analyses for appropriate ICD shock

	Univariate		Multivariate	
	HR (95%CI)	*P*-value	HR (95%CI)	*P*-value
Abnormal SAECG	2.56 (1.09–6.01)	0.031	2.67 (1.14–6.26)	0.024
Age (1 year increase)	1.00 (0.98–1.03)	0.907		
Male gender	1.74 (0.65–4.69)	0.272		
LVEF ≤ 30%	2.15 (0.80–5.78)	0.128		
NYHA class II	0.57 (0.13–2.49)	0.457		
eGFR < 60 mL/min/1.73 m^2^	2.48 (1.09–5.65)	0.030	2.61 (1.15–5.92)	0.021
Atrial fibrillation	1.63 (0.70–3.82)	0.258		
Beta-blocker use	1.02 (0.24–4.34)	0.983		
Digoxin use	1.20 (0.52–5.78)	0.128		
Amiodarone use	1.23 (0.54–2.79)	0.629		

*ACE* angiotensin-converting enzyme; *ARB* angiotensin II receptor blocker; *CI* confidence interval; *eGFR* estimated glomerular filtration rate; *HR* hazard ratio; *ICD* implantable cardioverter defibrillator; *LVEF* left ventricular ejection fraction; *MRA* mineralocorticoid receptor antagonist; *NYHA* New York heart association; *SAECG* signal-averaged electrocardiography

**Fig. 3:**
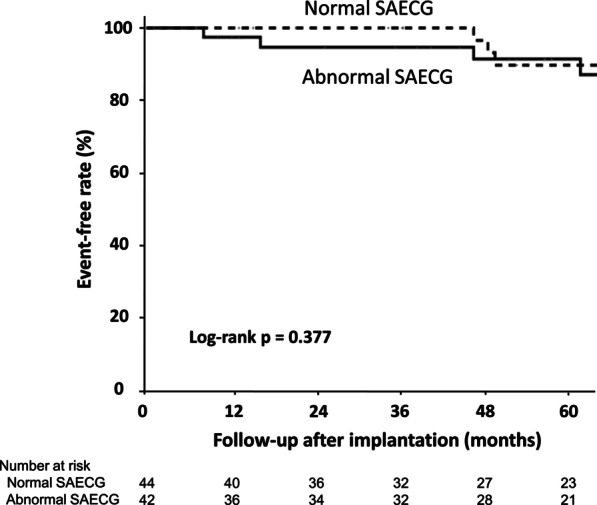
Kaplan–Meier curves for all-cause death in nonischemic systolic HF patients who received an ICD with abnormal SAECG findings and normal SAECG findings

Kaplan–Meier curves for all-cause death are depicted in Fig. 3. There was no difference in the incidence of all-cause death between patients with abnormal and normal SAECG findings. Among the patients who died, the most common cause of death was HF (Table 4).

**Table 4:** Causes of death

	Normal SAECG (n = 44)	Abnormal SAECG (n = 42)	*P*-value
Death from any cause	8	9	0.457
Cardiovascular death			0.232
SCD/arrhythmia	1	1	
Heart failure	4	8	
Noncardiovascular death	2	0	
Unknown	1	0	

*SAECG* signal-averaged electrocardiography; *SCD*: sudden cardiac death

## Discussion

Our study showed a higher incidence of appropriate shocks in patients with abnormal SAECG findings than in patients with normal SAECG findings among primary prophylactic ICD patients with nonischemic systolic HF. This relationship was observed in all patients and even in patients with a QRS complex < 120 ms. Abnormal SAECG findings were independently associated with the occurrence of appropriate shocks.

Abnormal SAECG findings denote the presence of depolarization abnormalities and an arrhythmic substrate in the ventricle, leading to the development of ventricular tachyarrhythmia [10]. In the 1990s, abnormal SAECG findings were reported to predict VT/VF and/or all-cause death in patients with nonischemic dilated cardiomyopathies [11, 12]. Since then, abnormal SAECG findings have been found to fail to predict death due to VT/VF, including appropriate ICD therapy or death, in patients with nonischemic dilated cardiomyopathies [10, 13, 19]. Overall, the treatment of HF, including beta-blocker therapy, has greatly progressed in the past two decades, and the rate of SCD has declined due to thorough evidence-based HF drug therapy development from the 1990s to 2000s [20]. Currently, it is difficult to distinguish high-risk patients from extensive HF patients using SAECG alone because of its low accuracy.

Recent progress in device programming has helped prevent the delivery of unnecessary ICD shocks. In this study, appropriate ICD shocks were less frequent than appropriate ATPs even in patients with both normal and abnormal SAECG findings. ICD shock delivery could be avoided with the ATP setting and delayed high-rate programming. Nevertheless, abnormal SAECG findings were a significant risk factor for appropriate shock therapy in high-risk patients with nonischemic HF who received primary prophylactic ICD in accordance with guideline recommendations. The incidence of appropriate shock therapy was higher in patients with abnormal SAECG findings than in those with normal SAECG findings, among whom most received renin-angiotensin system inhibitors and beta-blockers.

Previous reports have shown that a longer QRS duration is associated with increased risks for all-cause death, SCD and cardiac death in patients with HF [21]. In this study, in nonischemic HF patients with a QRS complex < 120 ms, there was a relationship between abnormal SAECG findings and appropriate ICD shock using conventional SAECG criteria. Approximately 20% of our patients had a QRS complex ≥ 120 ms. Most previous studies using SAECG excluded patients with a wide QRS complex, such as those with bundle branch block, and patients receiving ventricular pacing. A small study proposed modified SAECG criteria for identifying patients with induced or spontaneous VT among those with longer QRS complex durations (> 100 ms), although the majority of cases involved an ischemic etiology [22]. Another study proposed modified SAECG criteria, but the predictive values for SCD and cardiac death were low in nonischemic dilated cardiomyopathies despite the presence of a bundle branch block [23]. In clinical practice, however, it can be cumbersome to set different reference values for subjects with different clinical backgrounds. Therefore, for patients with a QRS complex ≥ 120 ms, we used conventional cutoff values for both the RMS40 and LAS40 parameters, excluding fQRS, and abnormal SAECG findings were defined if both parameters were abnormal in this study. Among patients with a QRS complex ≥ 120 ms, approximately 80% met the criteria for abnormal SAECG findings, and half of them experienced appropriate ICD shocks. In addition, the prolongation of the QRS duration reflects a left ventricular conduction delay, which is associated with left ventricular function and volume [21, 24] but indicates more than the focal late potential in HF patients. Therefore, it may be appropriate to define abnormal SAECG findings with two parameters, namely, RMS 40 and LAS 40, and exclude fQRS in patients with a QRS complex ≥ 120 ms.

The Coronary Artery Bypass Graft (CABG) Patch Trial, which included ischemic patients who underwent elective coronary bypass surgery and had a high risk for SCD, LVEF < 36% and abnormal SAECG findings, showed no survival benefit of implanting a prophylactic ICD at the time of surgery [25]. In the CABG Patch Trial, ICD therapy tended to reduce arrhythmic death (*p* = 0.057), but the differences in all-cause mortality did not reach significance because 71% of deaths were due to nonarrhythmic causes [26]. In our study, because approximately 90% of the deaths were due to nonarrhythmic causes, including HF and noncardiac causes, the presence of abnormal SAECG findings associated with life-threatening arrhythmia had little contribution to all-cause death. Therefore, abnormal SAECG findings alone might not affect all-cause death.

The complex interactions of an arrhythmogenic substrate with several triggers and modulators, such as autonomic tone, myocardial ischemia, electrolyte disturbance and worsening HF, contribute to the development of VT/VF. The late potential at the terminal part of the QRS complex may indicate the presence of a delay in myocardial conduction in the reentrant circuit as an arrhythmogenic substrate. The presence of an arrhythmogenic substrate is a predictor of VT/VF, and its disappearance can indicate a therapeutic effect on VT/VF. Previous reports have demonstrated that surgical or ablation treatment for VT normalizes SAECG findings and that patients with normalized SAECG findings after such procedures showed favorable outcomes compared to patients with persistent abnormal SAECG findings [27, 28]. Thus, the presence of ventricular late potential, which shows an arrhythmogenic substrate, may have a role in predicting the occurrence of VT/VF requiring ICD shocks in high-risk patients.

Among patients with abnormal SAECG findings, arrhythmogenic right ventricular cardiomyopathy or cardiac sarcoidosis was also present in a small number. This might be because patients with these cardiomyopathies are at increased risk and ventricular arrhythmias and SCD [29, 30] and because abnormal SAECG findings collectively serve as a useful diagnostic tool for detecting these cardiomyopathies [29, 31]. In this study, digoxin and amiodarone tended to be more frequently used among patients with abnormal SAECG findings than among patients with normal SAECG findings, but the use of both drugs did not affect the delivery of an appropriate shock. Although there has been no report that digoxin directly affects the SAECG findings, the use of digoxin may increase the frequency of ICD shocks [32]. On the other hand, amiodarone prolongs the fQRS and LAS 40 and decreases RMS 40, leading to abnormal SAECG findings [33], which have been reported to predict the effects of amiodarone in patients with myocardial infarction and VT [34]. Amiodarone might partially contribute to the expression of abnormal SAECG findings, but SAECG did not have enough power to predict the potential effects of drug treatments such as digoxin and amiodarone in high-risk patients with nonischemic HF.

In this study, an eGFR < 60 mL/min/1.73 m^2^, as well as abnormal SAECG findings, were independent predictive factors for ICD shock. Renal dysfunction is known to be a potential risk factor for SCD [35]. We previously reported that renal dysfunction with an eGFR < 60 mL/min/1.73 m^2^ was an independent predictor of appropriate ICD therapy in patients with nonischemic HF [36]. Identifying patients who are likely to receive appropriate shock and require management for the prevention of VT/VF is important for improving the survival of patients with HF and ICDs.

Currently, the most widely used criterion for implanting prophylactic ICDs in patients with HF is LVEF ≤ 35%, but there are certainly limitations in term of risk stratification. In 2016, the Danish Study to Assess the Efficacy of ICDs in Patients with Non-ischemic Systolic Heart Failure on Mortality (DANISH) showed that ICD implantation did not improve outcomes in patients with nonischemic HF and LVEF ≤ 35% [37], and the frequency of SCD has been decreasing as optimal HF medical therapies have become more thorough [20]. At present, the contribution of ICD therapy to improving outcomes in patients with systolic HF may diminish compared to the 2000s. Additionally, since ICDs also have negative risks, such as reduced quality of life, several complications associated with implantation/replacement, risk of device infection and inappropriate shocks, indications for ICDs must be more risk stratified. Therefore, identifying more accurate predictors of ventricular tachyarrhythmias in high-risk patients is needed. Although this is a small study from a single center, measuring SAECG values in addition to conventional risk factors may allow stratification of patients according to the risk for life-threatening arrhythmias associated with HF.

## Study limitations

There are some limitations in this study. First, this was a retrospective observational study conducted at a single center. Data concerning clinical conditions at the time of ICD therapy or death were not available, and treatment bias was present. Second, the diagnostic value of the RMS 40 and LAS 40 criteria in the ability of SAECG to detect the probability of SCD or death has not yet been established for patients with a wide QRS complex. However, the significant results in our patients with wide QRS complexes using conventional cutoff values for these SAECG parameters may be a useful reference in clinical practice, but these findings should be confirmed in other patient populations. Third, the number of patients was relatively small. Therefore, subgroup analyses were not feasible. Fourth, the ICD detection thresholds programmed for the VF zone and VT zone and ICD therapy settings, including anti-tachycardia pacing and shock, were not identical. Finally, during the long follow-up period of this study, a consensus on delayed high-rate programming was reached, and the use of this programming increased over the years. For this reason, we could not rule out the possibility that some unnecessary shocks that can be avoided by modern programming were partially counted as appropriate shocks in subjects in the 2000s.

## Conclusions

Our results suggest that abnormal SAECG findings are associated with a high risk of appropriate shocks in patients with prophylactic ICDs and nonischemic systolic HF.

## Supplementary Information

**Additional file 1: Table S1.** Device programing according to date of ICD implantation. **Table S2.** Number of appropriate ATPs/Shocks in patients who experienced ICD therapies. **Table S3.** Incidence of ICD shocks in patients with normal and abnormal SAECG findings.

## Data Availability

The datasets generated and/or analyzed during the current study are not publicly available due to privacy or ethical restrictions but are available from the corresponding author on reasonable request.

## Abbreviations

ATP: Antitachycardia pacing
CABG: Coronary artery bypass graft
DANISH: Danish study to assess the efficacy of ICDs in patients with non-ischemic systolic heart failure on mortality
eGFR: Estimated glomerular filtration rate
EU-CERT-ICD: European comparative effectiveness research to assess the use of primary prophylactic implantable cardioverter-defibrillators
fQRS: Filtered QRS duration
HF: Heart failure
ICD: Implantable cardioverter defibrillator
IVCD: Intraventricular conduction delay
LAS 40: Duration of low-amplitude potentials < 40 μV
LBBB: Left bundle branch block
L-IVCD: Intraventricular conduction delay with a left bundle branch block-predominant feature
LVEF: Left ventricular ejection fraction
NYHA: New York Heart association
O-IVCD: Intraventricular conduction delay with non-left bundle branch block-predominant feature
RBBB: Right bundle branch block
RMS 40: Root-mean-square voltage during the last 40 ms of the fQRS
SAECG: Signal-averaged electrocardiography
SCD: Sudden cardiac death
VF: Ventricular fibrillation
VT: Ventricular tachycardia
