# Supplementary material for: Impact of signal-averaged electrocardiography findings on appropriate shocks in prophylactic implantable cardioverter defibrillator patients with nonischemic systolic heart failure
Source: BMC Cardiovasc Disord. 2022 Aug 16;22:374. doi: 10.1186/s12872-022-02811-6 (PMC9382808; doi:10.1186/s12872-022-02811-6)
Supplement: Supplementary file 1 — Additional file 1: Table S1. Device programing according to date of ICD implantation. Table S2. Number of appropriate ATPs/Shocks in patients who experienced ICD therapies. Table S3. Incidence of ICD shocks in patients with normal and abnormal SAECG findings. [file 12872_2022_2811_MOESM1_ESM.pdf]

Impact of signal-averaged electrocardiography findings on appropriate shocks in prophylactic implantable cardioverter defibrillator patients with nonischemic systolic heart failure

Michiru Nomoto, Atsushi Suzuki, Tsuyoshi Shiga, Morio Shoda, Nobuhisa Hagiwara

Table S1. Device programing according to date of ICD implantation.

	2000-2006 n=26	2007-2012 n=36	2013-2018 n=24	P-value
Detection zones				0.001
Single zone (only VF zone)	10 (38)	3 (8)	1 (4)	
Multiple zones	16 (62)	33 (92)	23 (96)	
VF detection interval (bpm)	188 [182-188]	200 [188-214]	222 [222-222]	<0.001
Detection setting				
NID ≤ 18/24 or NID < 30/40 or < 20 intervals or < 2.5 sec	26 (100)	29 (81)	8 (33)	<0.001
NID > 18/24 or NID ≥ 30/40 or ≥ 20 intervals or ≥ 2.5 sec	0	7 (19)	16 (67)	
Fast VT detection zone set (n)	0	4 (11)	2 (8)	0.227
Fast VT detection interval (bpm)	—	176 [164-195]	191 [182-200]	0.404
VT detection zone set (n)	16 (62)	33 (92)	23 (96)	0.001
VT detection interval (bpm)	150 [150-160]	155 [148-169]	171 [150-171]	0.084
Detection setting				
NID ≤ 16 or ≤ 10 sec	15 (94)	13 (39)	1 (4)	<0.001
NID > 16 or > 10 sec	1 (6)	20 (61)	22 (96)	

Values are number (%) or median [interquartile range]. ICD, implantable cardioverter-defibrillator; NID, number of intervals to detect; VF, ventricular fibrillation; VT, ventricular tachycardia.

Continuous variables were compared among groups using the Kruskal-Wallis H nonparametric test. Categorical variables were subjected to chi-squared analysis.

The VF detection zone was programmed for all patients. The VT zone was programmed for 72 patients; 2 VT zones were programmed for 6 of these patients. Delayed high-rate programming has been used to avoid unnecessary shocks according to the guidelines and its use significantly increased over the years of the study period.

Table S2. Number of appropriate ATPs/Shocks in patients who experienced ICD therapies.

Normal SAECG			Abnormal SAECG		
Patient	ATP	Shock	Patient	ATP	Shock
1	116	2	1	93	8
2	58	0	2	65	1
3	29	1	3	51	1
4	17	0	4	28	1
5	14	1	5	23	2
6	5	11	6	17	1
7	5	0	7	13	4
8	3	0	8	13	0
9	2	0	9	10	2
10	1	0	10	8	1
11	1	0	11	3	2
12	0	7	12	3	0
13	0	7	13	2	0
14	0	5	14	1	1
15	0	2	15	1	0
			16	1	0
			17	0	13
			18	0	4
			19	0	3
			20	0	1
			21	0	1

ATP, antitachycardia pacing; SAECG, signal-averaged electrocardiography.

Appropriate ICD shocks were less frequent than appropriate ATPs for terminating ventricular tachyarrhythmias in both patients with normal and patients with abnormal SAECG findings.

Table S3. Incidence of ICD shocks in patients with normal and abnormal SAECG findings.

	Normal SAECG n=44			Abnormal SAECG n=42		
	Events	Patients	Median, per person	Events	Patients	Median, per person
Appropriate shocks						
Only counting the first shock	8			16		
All shocks	36	8	4	46	16	2
Appropriate ATP						
Only counting the first ATP	11			16		
All ATPs	251	11	5	332	16	12
Inappropriate shocks						
Only counting the first shock	8			11		
All shocks	15	8	1	52	11	3

ATP, antitachycardia pacing; CI, confidence interval; ICD, implantable cardioverter defibrillator; SAECG, signal-averaged electrocardiography.

The median numbers of appropriate shocks per patient was 4 and 2 in patients with normal and abnormal SAECG findings, respectively, the median numbers of appropriate ATPs per patient was 5 and 12 in patients with normal and abnormal SAECG findings, respectively, and the median numbers of inappropriate shocks per patient was 1 and 3 in patients with normal and abnormal SAECG findings, respectively.
